# Beta2-Adrenergic Receptor Polymorphisms and Haplotypes Associate With Chronic Pain in Sickle Cell Disease

**DOI:** 10.3389/fphar.2019.00084

**Published:** 2019-02-07

**Authors:** Ellie H. Jhun, Nilanjana Sadhu, Xiaoyu Hu, Yingwei Yao, Ying He, Diana J. Wilkie, Robert E. Molokie, Zaijie Jim Wang

**Affiliations:** ^1^Department of Biopharmaceutical Sciences, College of Pharmacy, University of Illinois at Chicago, Chicago, IL, United States; ^2^Department of Biobehavioral Health Science, College of Nursing, University of Illinois at Chicago, Chicago, IL, United States; ^3^Department of Biobehavioral Nursing Science, College of Nursing, University of Florida, Gainesville, FL, United States; ^4^Comprehensive Sickle Cell Center, University of Illinois at Chicago, Chicago, IL, United States; ^5^Jesse Brown Veteran’s Administration Medical Center, Chicago, IL, United States; ^6^Division of Hematology/Oncology, College of Medicine, University of Illinois at Chicago, Chicago, IL, United States

**Keywords:** single nucleotide polymorphism, haplotype, sickle cell disease, beta2-adrenergic receptor, chronic pain

## Abstract

Pain in sickle cell disease (SCD) is severe, variable, and inadequately comprehended. The β2-adrenergic receptor (*ADRB2*) is critical in mediating neurotransmitter response in the sympathetic nervous system. In this association study, we examined 16 single nucleotide polymorphisms (SNPs) covering 5′-UTR and coding regions of *ADRB2* for pain variability in SCD. Subjects recorded their non-crisis, baseline pain experience on a computerized tool from which we obtained chronic pain measurement score- composite pain index (CPI). Regression models yielded significant associations between chronic pain and seven SNPs. Non-synonymous SNP rs1042713 A allele (Arg16) caused a 5.73-fold decrease in CPI (*p* = 0.002). Allele A of rs12654778 and T of rs17778257 reduced CPI by a fold of 4.52 (*p* = 0.019), and 4.39 (*p* = 0.032), respectively. Whereas, in the 5′ UTR, allele C of rs1042711, G of rs11168070, C of rs11959427, and C of rs1801704 increased CPI by a fold of 10.86 (*p* = 0.00049), 5.99 (*p* = 0.016), 5.69 (*p* = 0.023), and 5.26 (*p* = 0.031), respectively. Together, these SNPs accounted for 2–15% of CPI variance after adjusting for covariates. Moreover, these SNPs were in high linkage disequilibrium (LD) showing three LD blocks in our cohort. A 10-marker haplotype increased CPI by 11.5-fold (*p* = 0.000407). Thus, *ADRB2* polymorphisms might contribute to chronic pain severity and heterogeneity in SCD.

## Introduction

Pain is a significant problem in patients with sickle cell disease (SCD) (Ballas et al., 2012). Not only is pain severe and lifelong, it is also highly heterogeneous which presents a challenge to effective treatment for all patients (Platt et al., 1991; Rees et al., 2010; Wilkie et al., 2010). SCD pain can be characterized as both acute and chronic pain (Smith et al., 2008; Wilkie et al., 2010; Ballas et al., 2012). The acute painful crisis is an unpredictable event that leads to emergency room care and hospitalization, and causes significant morbidity and mortality (Platt et al., 1991; Aisiku et al., 2009; Rees et al., 2010). The pain episode varies in frequency and severity as documented in a study of 29,922 SCD subjects where 29.4% of patients had no pain episode, but 16.9% had three or more crises annually (Brousseau et al., 2010). Persistent chronic pain is also found in patients with SCD and is highly heterogeneous. Smith et al. (2008) reported that 29.3% of patients reported pain in greater than 95% of self-reported pain diary days. In a study of SCD subjects at a routine outpatient clinic visit, mild pain intensity was reported in 17% of subjects, moderate pain in 27%, and severe pain in 19% (Wilkie et al., 2010). A more recent study found that at baseline, most SCD subjects reported ongoing mild pain (Campbell et al., 2015).

To understand pain heterogeneity in SCD, we examined the influence of candidate gene polymorphisms on pain in SCD. In this study, we focused on single nucleotide polymorphisms (SNPs) of the beta2-adrenergic receptor gene (*ADRB*2*)*. *ADRB2* codes for the β_2_-adrenergic receptor that is a member of the seven membrane-spanning G-protein coupled receptor superfamily and is a major receptor that mediates the responses of sympathetic neurotransmitters (Litonjua et al., 2010; Reiner et al., 2010). The β_2_-adrenergic receptor is expressed within the nociceptive system (Hein, 2006; Yalcin et al., 2010), including in the spinal cord superficial dorsal horn neurons (Nicholson et al., 2005), which is essential to pain transmission (Peng et al., 1993; Nicholson et al., 2005; Todd, 2010). Several *ADRB2* SNPs have been studied for pain in temporomandibular joint disorder (TMD) (Diatchenko et al., 2006) and chronic musculoskeletal complaints (Skouen et al., 2012). Three major haplotypes were found to be associated with *ADRB2* expression, psychological traits, resting arterial pressure, and development of TMD (Diatchenko et al., 2006). In the chronic musculoskeletal study, *ADRB2* rs2053044 and the H1-H1 haplotype showed an association with pain (Skouen et al., 2012). In the current study, we investigated 16 SNPs in the 5′-untranslated region (5′-UTR) and coding regions of the *ADRB2* gene, including several SNPs that have not been studied for pain (e.g., 5′-UTR rs1042711), on their influence on acute and chronic pain in patients with SCD.

## Materials and Methods

### Subjects

The University of Illinois at Chicago (UIC) Institutional Review Board approved the study. Blood and/or buccal swab samples were collected at the University of Illinois (UI) Hospital and Health Sciences System in Chicago, Chicago, IL, United States from patients during their regular clinic visits. All participants gave written informed consent. Analysis was conducted on 115 to 136 subjects with SCD where both clinical data and genetic samples were available. A power analysis was not performed *a priori* for this exploratory study.

### Genotyping and Pain Assessment

DNA was extracted from blood and buccal samples using a modified phenol/chloroform method, or a modified salting-out procedure, or the QuickGene DNA whole blood extraction kit as previously described (Jhun et al., 2014; Sadhu et al., 2018). This was followed by genotyping on the MassARRAY iPLEX Platform (Sequenom, San Diego, CA, United States) to generate genotype data. Chronic pain assessment utilized composite pain index (CPI) as a measure of the multidimensional pain experience. The reported CPI value has a range of 0 to 100 for each subject as previously reported for patients with SCD (Ezenwa et al., 2014). Briefly, the subjects recorded their baseline pain information on a computerized tool called PAINReportIt^®^ which is based on the well-established McGill Pain Questionnaire for pain assessment (Melzack, 1975). Self-reported baseline raw pain scores were converted to a scale of 0–100 and then averaged (Wilkie et al., 2003, 2010, 2015).

Alternatively, acute health care utilization served as the surrogate marker for acute crisis pain as reported by us previously (Ezenwa et al., 2014; Jhun et al., 2014; Sadhu et al., 2018). Utilization is defined as the number of admissions to the emergency department and/or acute care center resulting from a sickle cell pain crisis for the subsequent 12 months after the patient completed the baseline pain assessment. In short, data was collected by medical record review (for UI utilization) or biweekly telephones calls (for non-UI utilization).

### Statistical Analysis

Single nucleotide polymorphisms were selected based on literature as discussed in the introduction. Hardy–Weinberg equilibrium was evaluated by a χ^χ^ goodness-of-fit test. The effect of SNPs on CPI value was analyzed by additive (allele effects), dominant (major allele homozygous genotypes versus combined heterozygous and minor allele homozygous), and recessive (combined major allele homozygous genotype and heterozygous versus minor allele homozygous) multiple linear regression models (Lettre et al., 2007; Clarke et al., 2011) adjusted for age, sex, ethnicity, and sickle cell type with major alleles as reference genotypes in the analysis. SNP effects on utilization data was analyzed by additive, dominant, and recessive negative binomial regression models (Cameron and Trivedi, 1998) adjusted for age, sex, ethnicity, and sickle cell type. SNP effects on three different utilization groups were analyzed by an additive, dominant, and recessive ordinal logistic regression model adjusted for the same covariates (Ezenwa et al., 2014). The regression models used to fit the data were driven by the nature and distribution of the dependent variables- CPI and utilization. Zero, low, and high utilization categories for logistic regression were based on previous work by us and others (Epstein et al., 2006; Lanzkron et al., 2006; Carroll et al., 2011). The recessive model for the following SNPs was not performed since the minor allele frequency was too low: rs11168070, rs11959427, rs1042711, and rs1801704. Analysis was performed on SPSS software (version 20; IBM, Armonk, NY, United States) or on R (version 3.4.0; R Foundation for Statistical Computing, Vienna, Austria) for these analyses. Linkage disequilibrium (LD) plot was generated from Haploview version 4.2 (Broad Institute, Cambridge, MA, United States) (Barrett et al., 2005). Haplotype association analyses were performed with PLINK (Massachusetts General Hospital and the Broad Institute of Harvard and MIT, Cambridge) (Purcell et al., 2007; Purcell, 2010) Hap-linear options were used on PLINK to include covariates (age, sex, ethnicity, and sickle cell type).

## Results

Patient demographics for the 136 subjects are provided in Table 1. The average age of the SCD subjects in our cohort was 34 years with a range from 15 to 70 years and a median of 32 years. More females than males were enrolled in the study; however, no known preference was given toward females during recruitment and the prevalence of SCD is not known to be gender biased. In SCD, males have been reported to have more frequent admissions for pain crises (Ballas, 2005), and higher mortality than females (Shankar et al., 2005). Sickle cell types and self-reported ethnicity are also given in Table 1. The mean CPI (Wilkie et al., 2015), a measurement for chronic pain, was 40.4 with a large range of 14.8 to 86.5, reaffirming pain heterogeneity. Utilization within a period of 12 months, another highly variable pain phenotype, ranged from 0 to 38. The number of subjects within each utilization group is also given and separated into no, low (Platt et al., 1991; Wilkie et al., 2010; Ballas et al., 2012) and high (>3) groups based on previous studies (Platt et al., 1991; Brousseau et al., 2010; Ezenwa et al., 2014).

**Table 1:** Patient Demographics (*N* = 136).

Age	Mean ± SD	34.0 ± 11.7
	Minimum	15
	Maximum	70
Sex, *n* (%)	Female	89 (65)
	Male	47 (35)
Ethnicity, *n* (%)	African American	132 (97)
	Hispanic	3 (2)
	Caucasian	1 (1)
Sickle cell type^∗^, *n* (%)	SCD-SS	105 (77)
	SCD-SC	15 (11)
	SCD-Sβ^+^	8 (6)
	SCD-Sβ°	7 (5)
	SCD-Sα	1 (1)
CPI	Mean ± SD	40.4 ± 13.5
	Minimum	14.8
	Maximum	86.5
Utilization	Mean ± SD	4.4 ± 5.2
	Median	3
	Lower quartile–upper quartile	1–5
	Minimum	0
	Maximum	38
Utilization groups, *n* (%)	Zero (0)	19 (14)
	Low (1–3)	60 (44)
	High (4–38)	57 (42)


*^∗^Sickle cell types: SCD-SS (sickle cell disease-homozygous hemoglobin S, sickle cell anemia), SCD-SC (sickle cell disease-sickle hemoglobin C), SCD-Sβ^+^ (sickle cell disease-sickle β^+^ thalassemia), SCD-Sβ° (sickle cell disease-sickle β° thalassemia), SCD-Sα (sickle cell disease-sickle α thalassemia).*

Allele and genotype frequencies for all 16 SNPs are listed in Table 2. SNPs are listed in order of chromosomal location from 5′→3′ starting from 5′-UTR. No significant deviations from Hardy–Weinberg equilibrium were observed for any of the 16 SNPs (*p* > 0.05).

**Table 2:** Allele and genotype frequencies.

dbSNP ID	Chromosome position^∗^	Allele, *n* (%)		Genotype, *n* (%)		
		Major	Minor	Major homozygote	Heterozygote	Minor homozygote
11958940	148821922	T, 127 (56.2)	A, 99 (43.8)	35 (31.0)	57 (50.4)	21 (18.6)
1432622	148824199	C, 129 (57.1)	T, 97 (42.9)	36 (31.9)	57 (50.4)	20 (17.7)
17778257	148825014	A, 185 (77.1)	T, 55 (22.9)	73 (60.8)	39 (32.5)	8 (6.7)
2895795	148825403	T, 167 (67.9)	A, 79 (32.1)	59 (48.0)	49 (39.8)	15 (12.2)
2400707	148825489	G, 128 (56.1)	A, 100 (43.9)	36 (31.6)	56 (49.1)	22 (19.3)
2053044	148825809	G, 153 (56.7)	A, 117 (43.3)	44 (32.6)	65 (48.1)	26 (19.3)
12654778	148826178	G, 204 (76.1)	A, 64 (23.9)	78 (58.2)	48 (35.8)	8 (6.0)
11168070	148826364	C, 222 (82.8)	G, 46 (17.2)	90 (67.2)	42 (31.3)	2 (1.5)
11959427	148826465	T, 217 (82.8)	C, 45 (17.2)	88 (67.2)	41 (31.3)	2 (1.5)
1042711	148826785	T, 251 (92.3)	C, 21 (7.7)	117 (86.0)	17 (12.5)	2 (1.5)
1801704	148826812	T, 217 (82.2)	C, 47 (17.8)	87 (65.9)	43 (32.6)	2 (1.5)
1042713	148826877	G (Gly), 116 (50.9)	A (Arg), 112 (49.1)	29 (25.4)	58 (50.9)	27 (23.7)
1042717	148827083	G (Leu), 151 (66.8)	A (Leu), 75 (33.2)	54 (47.8)	43 (38.1)	16 (14.2)
1042718	148827354	C (Arg), 178 (67.9)	A (Arg), 84 (32.1)	64 (48.9)	50 (38.2)	17 (13.0)
1042719	148827884	G (Gly), 165 (66.5)	C (Gly), 83 (33.5)	57 (46.0)	51 (41.1)	16 (12.9)
1042720	148828070	A (Leu), 147 (54.0)	G (Leu), 125 (46.0)	42 (30.9)	63 (46.3)	31 (22.8)


*dbSNP IDs are from the National Center for Biotechnology Information database (NCBI). Gly, glycine; Arg, arginine; Leu, leucine. ^∗^Chromosome position is from NCBI (National Center for Biotechnology Information) GRCh38 Assembly.*

Seven of the 16 *ADRB2* SNPs were found to be significantly associated with CPI in the additive multiple linear regression model adjusted for age, sex, ethnicity, and sickle cell type (Table 3). 5′-UTR rs1042711 C allele, rs11168070 G allele, rs11959427 C allele, and rs1801704 C allele were associated with increased CPI of 10.86 (*p* = 0.00049), 5.99 (*p* = 0.016), 5.69 (*p* = 0.023), and 5.26 (*p* = 0.031), respectively (Table 3). On the other hand, coding SNP rs1042713 A allele (Arg16), rs17778257 T allele, and rs12654778 A allele were associated with CPI reduction of 5.73 (*p* = 0.002), 4.39 (*p* = 0.032), and 4.52 (*p* = 0.019), respectively.

**Table 3:** Multiple linear regression models evaluating the effects of *ADRB2* SNPs on CPI.

dbSNP ID	Model	B (95% CI)^∗^	*p*-Value	Adj. R^χ†^
11958940	Add	3.00 (–0.67, 6.68)	0.11	0.09
	Rec	4.82 (–1.83, 11.47)	0.15	0.09
	Dom	3.37 (–2.13, 8.86)	0.23	0.08
1432622	Add	3.14 (–0.58, 6.86)	0.10	0.09
	Rec	4.41 (–2.41, 11.22)	0.20	0.08
	Dom	3.88 (–1.57, 9.33)	0.16	0.08
17778257	Add	–4.39 (–8.40, –0.38)	**0.03**	0.02
	Rec	–4.26 (–14.19, 5.68)	0.40	–0.01
	Dom	–5.98 (–11.09, –0.87)	**0.02**	0.03
2895795	Add	1.52 (–2.16, 5.20)	0.41	0.05
	Rec	0.91 (–6.47, 8.30)	0.81	0.04
	Dom	2.49 (–2.60, 7.57)	0.33	0.05
2400707	Add	2.56 (–1.08, 6.21)	0.17	0.08
	Rec	3.89 (–2.68, 10.45)	0.24	0.07
	Dom	3.03 (–2.42, 8.49)	0.27	0.07
2053044	Add	2.45 (–0.94, 5.84)	0.15	0.01
	Rec	5.51 (–0.46, 11.49)	0.07	0.02
	Dom	1.58 (–3.55, 6.71)	0.54	0.00
12654778	Add	–4.52 (–8.28, –0.75)	**0.02**	0.04
	Rec	–4.63 (–14.34, 5.08)	0.35	0.00
	Dom	–5.87 (–10.54, –1.20)	**0.01**	0.04
11168070	Add	5.99 (1.12, 10.85)	**0.02**	0.04
	Rec	N/A^∗∗^	N/A	N/A
	Dom	5.67 (0.58, 10.76)	**0.03**	0.03
11959427	Add	5.69 (0.82, 10.56)	**0.02**	0.05
	Rec	N/A	N/A	N/A
	Dom	5.34 (0.23, 10.46)	**0.04**	0.05
1042711	Add	10.86 (4.85,16.86)	**<0.001**	0.09
	Rec	N/A	N/A	N/A
	Dom	11.28 (4.74, 17.81)	**0.001**	0.08
1801704	Add	5.26 (0.49, 10.02)	**0.03**	0.03
	Rec	N/A	N/A	N/A
	Dom	4.89 (–0.10, 9.87)	0.055	0.02
1042713	Add	–5.73 (–9.24, –2.23)	**0.002**	0.15
	Rec	–8.53 (–14.33, –2.72)	**0.004**	0.13
	Dom	–6.62 (–12.38, –0.86)	**0.02**	0.10
1042717	Add	1.96 (–1.63, 5.55)	0.28	0.08
	Rec	3.51 (–3.62, 10.64)	0.33	0.08
	Dom	2.23 (–2.97, 7.43)	0.40	0.07
1042718	Add	0.89 (–2.74, 4.51)	0.63	–0.01
	Rec	0.74 (–6.49, 7.97)	0.84	–0.01
	Dom	1.39 (–3.72, 6.49)	0.59	–0.01
1042719	Add	–0.42 (–4.20, 3.36)	0.83	–0.02
	Rec	0.38 (–7.13, 7.90)	0.92	–0.02
	Dom	–1.01 (–6.28, 4.26)	0.71	–0.02
1042720	Add	–0.29 (–3.72, 3.13)	0.87	0.00
	Rec	–1.35 (–7.41, 4.71)	0.66	0.00
	Dom	0.31 (–4.83, 5.44)	0.91	0.00


*^∗^Unstandardized regression coefficient and 95% confidence interval. **^†^**Adjusted R-square for five predictors (SNP, age, sex, ethnicity, and sickle cell type). ^∗∗^Minor allele frequency is too low for the analysis in a recessive model for these SNPs: rs11168070, rs11959427, rs1042711, and rs1801704. Add, additive; Dom, dominant; Rec, recessive regression models were used. The major alleles are the reference genotypes in the analyses. Significance level of bold values are 0.05.*

In the dominant regression models, rs17778257 TT/TA, rs12654778 AA/AG, rs1042713 AA/AG genotypes were associated with CPI reduction of 5.98 (*p* = 0.022), 5.87 (*p* = 0.014), and 6.62 (*p* = 0.025), respectively. SNPs rs11168070 GG/GC, rs11959427 CC/CT, and rs1042711 CC/CT genotypes, on the other hand, associated with CPI increase of 5.67 (*p* = 0.029), 5.34 (*p* = 0.041), and 11.28 (*p* = 0.001), respectively. Rs1801704 did not show statistical significance in the dominant model (*B* = 4.89, 95%CI [-0.10, 9.87], *p* = 0.055).

**FIGURE 1:**
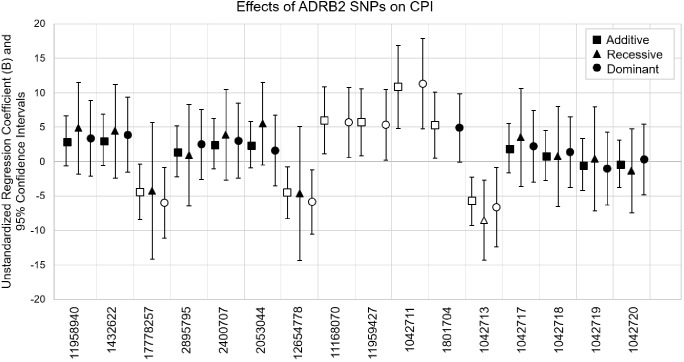
Multiple linear regression models evaluating the effects of *ADRB2* SNPs on CPI. SNPs are in the order of *ADRB2* 5′→3′. Unstandardized regression coefficients with 95% confidence intervals for additive, recessive and dominant models are shown for each SNP. The major alleles are the reference genotypes in the analyses. Open symbols are significant associations (*p* < 0.05).

Coding SNP rs1042713 was the only SNP to show significance in all three models including the recessive regression model where the AA genotype caused 8.53-fold decrease in CPI (*p* = 0.004). These models explained 2–15% of the variance in CPI (adjusted r-square). A figure with unstandardized regression coefficients for each SNP and model is shown in Figure 1.

We performed association analyses in African American only cohort (n = 132). Findings were consistent. In the additive model risk alleles of rs1042711, rs11168070, rs11959427, and rs1801704 associated with increased CPI and that of rs1042713, rs17778257, and rs12654778 with decreased CPI. Similarly, in the dominant model, risk alleles of rs17778257, rs12654778, and rs1042713 associated with CPI reduction and that of SNPs rs11168070, rs11959427, and rs1042711 with increase in CPI. SNP rs1042713 too exhibited significant association with decreased CPI in recessive model. Additionally, minor alleles of SNPs rs1432622 (*p* = 0.037) and rs11958940 (*p* = 0.043) in the additive model, rs1801704 (*p* = 0.039) in the dominant model, and rs2053044 (*p* = 0.048) in the recessive also showed significant association with increase in CPI.

We did not find any significant influences of *ADRB2* SNPs on the number of utilization or acute pain in the 136 SCD subjects (Table 4) or in the African American only cohort.

**Table 4:** Negative binomial regression model evaluating the effects of *ADRB2* SNPs on utilizations.

dbSNP ID	Model	IRR (95% CI)^∗^	*p*-Value
11958940	Add	1.02 (0.77, 1.35)	0.89
	Rec	0.87 (0.54, 1.42)	0.58
	Dom	1.15 (0.76, 1.74)	0.50
1432622	Add	0.96 (0.72, 1.27)	0.75
	Rec	0.83 (0.50, 1.37)	0.46
	Dom	1.03 (0.68, 1.55)	0.90
17778257	Add	0.93 (0.69, 1.25)	0.63
	Rec	0.77 (0.37, 1.58)	0.47
	Dom	0.96 (0.66, 1.38)	0.81
2895795	Add	1.01 (0.75, 1.37)	0.93
	Rec	0.84 (0.47, 1.49)	0.55
	Dom	1.11 (0.75, 1.67)	0.60
2400707	Add	1.00 (0.75, 1.34)	0.10
	Rec	0.81 (0.49, 1.33)	0.40
	Dom	1.16 (0.77, 1.77)	0.48
2053044	Add	0.99 (0.77, 1.28)	0.96
	Rec	1.03 (0.66, 1.60)	0.91
	Dom	0.97 (0.66, 1.42)	0.86
12654778	Add	0.95 (0.71, 1.28)	0.73
	Rec	0.77 (0.36, 1.61)	0.48
	Dom	0.98 (0.69, 1.41)	0.93
11168070	Add	1.17 (0.82, 1.67)	0.38
	Rec	N/A^∗∗^	N/A
	Dom	1.16 (0.79, 1.69)	0.45
11959427	Add	1.17 (0.82, 1.68)	0.38
	Rec	N/A	N/A
	Dom	1.16 (0.79, 1.69)	0.45
1042711	Add	1.41 (0.91, 2.19)	0.13
	Rec	N/A	N/A
	Dom	1.43 (0.89, 2.32)	0.14
1801704	Add	1.13 (0.79, 1.61)	0.52
	Rec	N/A	N/A
	Dom	1.10 (0.75, 1.62)	0.61
1042713	Add	0.90 (0.67, 1.20)	0.47
	Rec	0.76 (0.47, 1.23)	0.26
	Dom	0.98 (0.63, 1.52)	0.92
1042717	Add	1.05 (0.79, 1.41)	0.72
	Rec	0.75 (0.44, 1.29)	0.29
	Dom	1.29 (0.87, 1.92)	0.21
1042718	Add	1.05 (0.79, 1.39)	0.75
	Rec	0.73 (0.42, 1.24)	0.24
	Dom	1.26 (0.87, 1.84)	0.23
1042719	Add	1.00 (0.75, 1.33)	0.98
	Rec	0.80 (0.46, 1.39)	0.43
	Dom	1.11 (0.75, 1.63)	0.62
1042720	Add	1.04 (0.80, 1.35)	0.76
	Rec	1.02 (0.64, 1.62)	0.95
	Dom	1.08 (0.74, 1.58)	0.69


*^∗^Incident rate ratio and 95% confidence interval. ^∗∗^Minor allele frequency is too low for the analysis in a recessive model for these SNPs: rs11168070, rs11959427, rs1042711, and rs1801704. Add, additive; Dom, dominant; Rec, recessive regression models were used and adjusted for age, sex, ethnicity, and sickle cell type. The major alleles are the reference genotypes in the analyses.*

Previously, we found CPI scores predicted subsequent 1-year acute care utilization that can be divided into three groups: none (zero), 1 to 3 (low), or > 3 events (high) (Ezenwa et al., 2014). We took a similar approach and separated our data into three groups: none (zero), 1 to 3 (low), or > 3 events (high) for analyses with covariates that included age, sex, ethnicity, and sickle cell type in order to identify independent effects of SNPs on utilization. Again, ordinal logistic regression models did not reveal significant influence of *ADRB2* SNPs on utilization (Table 5). These data suggested that *ADRB2* SNPs influence chronic, but not acute, pain in SCD.

**Table 5:** Ordinal logistic regression models evaluating effects of *ADRB2* SNPs on utilization.

dbSNP ID	Model	Estimate (95% CI)^∗^	*p*-Value
11958940	Add	0.13 (–0.41, 0.67)	0.63
	Rec	0.40 (–0.58, 1.38)	0.42
	Dom	0.02 (–0.78, 0.81)	0.96
1432622	Add	0.03 (–0.52, 0.57)	0.92
	Rec	0.25 (–0.75, 1.24)	0.62
	Dom	–.0.11 (–0.90, 0.69)	0.79
17778257	Add	–0.16 (–0.74, 0.41)	0.58
	Rec	0.52 (–0.95, 1.99)	0.49
	Dom	–0.40 (–1.13, 0.33)	0.29
2895795	Add	–0.07 (–0.60, 0.46)	0.79
	Rec	–0.06 (–1.12, 0.99)	0.91
	Dom	–0.11 (–0.84, 0.62)	0.77
2400707	Add	0.08 (–0.45, –0.60)	0.77
	Rec	0.23 (–0.72, 1.17)	0.64
	Dom	0.02 (–0.76, 0.80)	0.96
2053044	Add	0.07 (–0.42, 0.55)	0.79
	Rec	0.34 (–0.52, 1.21)	0.44
	Dom	–0.10 (–0.82, 0.63)	0.79
12654778	Add	–0.19 (–0.74, 0.36)	0.50
	Rec	0.60 (–0.89, 2.09)	0.43
	Dom	–0.43 (–1.11, 0.26)	0.22
11168070	Add	0.24 (–0.46, 0.95)	0.49
	Rec	N/A**	N/A
	Dom	0.18 (–0.55, 0.91)	0.62
11959427	Add	0.21 (–0.50, 0.92)	0.56
	Rec	*N/A*	N/A
	Dom	0.15 (–0.59, 0.88)	0.70
1042711	Add	0.58 (–0.34, 1.51)	0.21
	Rec	*N/A*	N/A
	Dom	0.53 (–0.46, 1.51)	0.29
1801704	Add	0.15 (–0.55, 0.85)	0.67
	Rec	*N/A*	N/A
	Dom	0.08 (–0.65, 0.80)	0.83
1042713	Add	–0.22 (–0.75, 0.31)	0.42
	Rec	–0.27 (–1.13, 0.60)	0.55
	Dom	–0.30 (–1.15, 0.55)	0.49
1042717	Add	0.05 (–0.47, 0.57)	0.85
	Rec	–0.17 (–1.20, 0.85)	0.74
	Dom	0.20 (–0.55, 0.95)	0.60
1042718	Add	0.10 (–0.41, 0.61)	0.70
	Rec	–0.06 (–1.06, 0.95)	0.91
	Dom	0.23 (–0.48, 0.94)	0.52
1042719	Add	0.09 (–0.43, 0.61)	0.74
	Rec	–0.07 (–1.10, 0.97)	0.90
	Dom	0.19 (–0.53, 0.92)	0.61
1042720	Add	–0.10 (–0.58, 0.38)	0.68
	Rec	–0.02 (–0.87, 0.83)	0.96
	Dom	–0.21 (–0.94, 0.51)	0.57


*^∗^Ordered log-odds estimate and 95% confidence interval. ^∗∗^Minor allele frequency is too low for the analysis in a recessive model for these SNPs: rs11168070, rs11959427, rs1042711, and rs1801704. Ordered log-odds estimate that the minor allele would result in a higher utilization group. Add, additive; Dom, dominant; Rec, recessive models were used and adjusted for age, sex, ethnicity, and sickle cell type. The major alleles are the reference genotypes in the analyses.*

**FIGURE 2:**
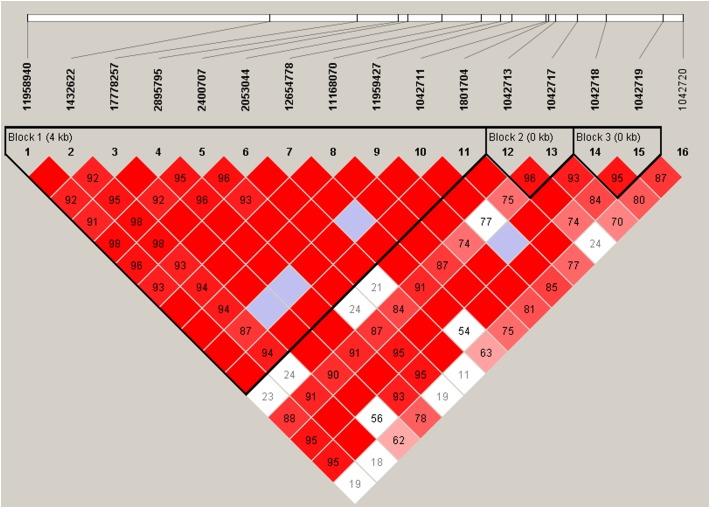
Linkage disequilibrium plot of *ADRB2* SNPs. Haplotype block organization of 16 *ADRB2* SNPs generated from Haploview 4.2 using the standard color scheme with the plot displaying D′ values, i.e., the linkage disequilibrium coefficients. Red = high D′ and high LOD, white = low D′, and low LOD; blue = high D′ and low LOD; shades of pink/red = low D′ and high LOD. Haplotype blocks did not change when four non-African American subjects were excluded.

*ADRB2* SNPs have previously been reported to be a part of a nine-marker haplotype where 2–4 markers were sufficient to maximize haplotype diversity (Belfer et al., 2005). We analyzed our SNPs for linkage disequilibrium (LD) using Haploview (Figure 2). Three haplotype blocks were formed from 15 SNPs (block 1: 11958940, 1432622, 17778257, 2895795, 2400707, 2053044, 12654778, 11168070, 11959427, 1042711, 1801704; block 2: 1042713, 1042717; and block 3: 1042718, 1042719).

These haplotypes (Table 6) were able to capture > 96% of the haplotypes from block 1, >99% from block 2, and >99% from block 3. Haplotype E consisting of markers 1–11 from block 1 (annotated in Figure 2) associated with an 11.5-fold increase in CPI score (*p* = 0.0004, Table 6). Haplotype E differs from D by rs1042711 that contains the C allele that increased CPI by 10.86 (Table 3, *p* = 0.0004). Other haplotypes containing the T allele, which decreased CPI by 4.29 in haplotype B (*p* = 0.033). Block 2 haplotypes AG caused 6.03 decrease in CPI score (*p* = 0.001) and GG increase CPI by 6.49 (*p* = 0.009). GA haplotype did not significantly associate with CPI. Rs1042713 may have a major role in influencing CPI score in this block, however, rs1042717 cannot be discounted as GA haplotype was not significant. Haplotype CC in block 3 approached statistical significance with a reduced CPI of 8.67 (*p* = 0.052). Haplotype analyses with African American only subjects yielded same haploblocks and pain-associated haplotypes.

**Table 6:** Haplotype frequencies and CPI association analyses.

Block (marker #)^∗^	Haplotype name	Haplotype	Frequency (%)	*B*	*p*-Value
Block 1 (1–11)	A	TCAAGGGCTTT	32.1	0.79	0.65
	B	TCTTGGACTTT	23.7	–4.29	**0.03**
	C	ATATAAGCTTT	24.8	–0.82	0.69
	D	ATATAAGGCTC	8.7	–1.23	0.71
	E	ATATAAGGCCC	7.0	11.50	**<0.001**
Block 2 (12, 13)		GA	32.9	2.14	0.24
		AG	48.1	–6.03	**0.001**
		GG	18.4	6.49	**0.009**
Block 3 (14, 15)		AC	29.6	1.20	0.56
		CC	3.8	–8.67	0.052
		CG	65.8	0.81	0.68


*^∗^Marker numbers are from Figure 2 LD plot. *p*-Values in bold are significant. Association analyses are performed with PLINK using the hap-linear option to include covariates (age, sex, ethnicity, sickle cell type).*

## Discussion

The beta2-adrenergic receptor (β_2_-AR) is a major receptor that mediates the action of sympathetic neurotransmitters NE and E. The *ADRB2* gene polymorphisms had not been studied for pain in SCD. Here, we showed that *ADRB2* SNPs and haplotypes have an important role in influencing chronic, but not acute, pain in SCD. Seven of the 16 SNPs examined (rs1042711, rs1042713, rs12654778, rs17778257 C, rs11168070, rs11959427, and rs1801704) were found to be associated with the severity of chronic pain, accounting for 2–15% of the variance in CPI after adjusting for variables including age, sex, ethnicity, and sickle cell type. *Post hoc* power analyses using statistical tool, G^∗^Power, indicated that our study has approximately 85% power to detect the observed effect sizes at a significance level of α = 0.05 (Faul et al., 2007). Furthermore, haplotype analysis found that several of these SNPs are in LD. We found the 10-marker haplotype caused 11.5 points increase in CPI (*p* = 0.0004). The coding region SNP rs1042711 alone was associated with an increase of more than 10 points in CPI. Although we found that CPI and age were significant predictors of utilization events (Ezenwa et al., 2014), none of the *ADRB2* SNPs showed significant associations with utilization.

The β_2_-AR has been known to play a pivotal role in pain perception and transmission. Agonists of β_2_-AR have been shown to attenuate chronic pain in rodent models of experimental mononeuropathy (Choucair-Jaafar et al., 2009, 2011), and diabetic peripheral neuropathy (Choucair-Jaafar et al., 2014; Baraka et al., 2015). Reductions in β_2_-ARs are associated with psychiatric disorders and comorbidities (Diatchenko et al., 2006) and stimulation of central β_2_-ARs produced antidepressant-like effects (Zhang et al., 2003). The converse was true where antidepressants recruitment of noradrenaline and its stimulation of β_2_-AR resulted in the relief of allodynia (Bohren et al., 2013).

The β_2_-AR also interacts with other receptors in modulating pain. For example, pancreatic hyperalgesia induced by sensitization of purinergic receptors was found to be mediated by adrenergic signaling in primary sensory neurons and attenuated by blocking the purinergic receptor or β_2_-AR (Wang et al., 2015).

In addition, β_2_-AR downstream signaling pathways of the beta2-adrenergic receptor have also been associated with pain. Hyperalgesia induced by epinephrine was prolonged by low levels of G protein-coupled receptor kinase 2 (GRK2) (Wang et al., 2011). GRK2 phosphorylates epinephrine activated β_2_-ARs. Furthermore, heterotypical intermittent stress-treated rats showed visceral hyperalgesia that was alleviated by β_2_-AR antagonist but not β_1_- or β_3_-AR antagonists (Zhang et al., 2014). β_2_-AR antagonists also block the hyperalgesic effects of opioids (Samoshkin et al., 2015). Moreover, β_2_-AR modulates opioid tolerance and physical dependence as shown in a study where morphine failed to cause tolerance in β_2_-AR knockout mice and physical dependence was reduced (Liang et al., 2007). Endogenous agonists of β_2_-AR show distinct conformational changes that can lead to different downstream signaling effects (Reiner et al., 2010).

The coding region rs1042713 is a non-synonymous SNP (Gly16Arg) that has been previously studied in affecting expression and function of β_2_-AR (Green et al., 1994, 1995; Small et al., 2003). *In vitro* studies found that rs1042713 Gly16 enhanced agonist-induced receptor downregulation (Green et al., 1994; Small et al., 2003). Haplotypes containing homozygous Arg16 allele were associated with the highest temporomandibular disorder incidence rate (Diatchenko et al., 2006). Another haplotype combination including Arg16 allele was found to be a risk factor for fibromyalgia syndrome (Vargas-Alarcon et al., 2009). In chronic widespread pain, however, Gly16 allele was associated with an increased risk for the disorder (Hocking et al., 2010). On the other hand, another study did not find any association of migraine with rs1042713 genotype, allele or haplotype (Schurks et al., 2009).

In our studies, we found that rs1042713 allele and genotype were associated with chronic pain in SCD using three different regression models. The Arg16 allele associated with lower chronic pain. Our finding is in agreement with attenuation of (chronic) pain by β_2_-AR agonists (Green et al., 1994). Patients with 16Arg would have less agonist-induced downregulation of the receptor. Moreover, Gly16 may directly affect SCD. In a study examining sickle red cell adhesion to laminin, Gly16 homozygotes showed significantly higher measurements of adhesion than the heterozygous and Arg16 homozygous genotypes combined (Eyler et al., 2008). This increased adhesion in sickle red blood cells may lead to increased disease severity in SCD. This is in line with our finding that Arg16 allele had strong association with lower baseline pain in our SCD population. These results suggest that rs1042713 plays a major role in SCD pain reported during a routine outpatient clinic visit. Of the 16 SNPs investigated rs1042717, rs1042718, rs1042719, and rs1042720 lie in the exon region of the gene along with rs1042713. They are reported to be synonymous variants and functional roles have not been established. It is thereby important to understand their contribution to linkage disequilibrium.

Although rs1042713 plays a significant role in baseline pain, other SNPs in linkage disequilibrium with this haploblock could be contributing to baseline pain in SCD as well (Belfer et al., 2005; Diatchenko et al., 2006). LD plot and haplotype analysis with rs1042717 revealed that rs1042713 risk allele was not always significantly associated with CPI. The GA haplotype in block 2 (rs1042713–rs1042717) consisting of rs1042713 Gly16 allele and A (Leu84) allele of rs1042717 did not associate with CPI. GG haplotype, however, caused a 5.88 increase in CPI score (*p* = 0.013). AG haplotype caused a decrease in CPI score. On the other hand, though rs1042717 is not independently associated with CPI scores as seen in Table 3, but as part of the rs1042713-rs1042717 haploblock we find that certain haplotypes associate with CPI. Conflicting conclusions from other studies also suggest the influence of haplotypes in different pain phenotypes. *ADRB2* rs11958940, rs1432622, rs2400707, rs1042717, and rs1042719 were found to be in linkage disequilibrium with rs1042713 in African Americans forming one haploblock (Belfer et al., 2005). Our LD plots however show two separate blocks with the lesser number of SNPs.

*ADRB2* rs1042711 lies in the 5′-untranslated region and has been studied in haplotype analysis for a malaria and asthma study where the T allele was shown to be both protective and associated with risk for malaria and asthma (Saadi et al., 2013). The other SNPs in this study included rs1801704, rs1042713, rs1042714, rs1042717, and rs1042718. Perhaps the SNPs that were not genotyped for our study would have had an influence over the effects of rs1042711 C allele in our study as associations seen in the malaria and asthma study show significant influences by other SNPs. A study on childhood lung function and *ADRB2* haplotypes show that a haplotype with the rs1042711 C allele was possibly showing a protective effect on reduced FEV_1_ (forced expiratory volume at 1-s) in 10-year old children (Torjussen et al., 2013). The other SNPs included in this study were rs1042713, rs1042714, and rs1800888. The latter two lie in the exon region as well with rs1042714 being a stop-gained variant and rs1800888 being a non-synonymous variant. *ADRB2* rs1042711 has not been previously studied for pain. The C allele in our study increases CPI score and the SNP is also in high LD with other SNPs but may confer a greater influence on CPI over the effect of the whole haplotype.

The findings of this study are limited by the small sample size and need to be replicated in larger studies that also evaluate the potential effect of population structure and admixture on the association. It would have also been of interest to examine if hydroxyurea has any effect on the observed associations. Unfortunately, we do not have information on hydroxyurea use at the time of sample acquisition. However, our center had contributed a large number of subjects to the hydroxyurea study and patients who tolerated therapy continued on it. While there has been some speculation on the role of hydroxyurea in decreasing acute care utilization (Lanzkron et al., 2018; Zhou et al., 2018), we were not able to add to these findings.

Our study identifies β2-AR as a potential target in the development of pharmacological agents for alleviating chronic baseline pain in SCD.

## Ethics Statement

This study was carried out in accordance with the recommendations of the University of Illinois at Chicago (UIC) Institutional Review Board, with written informed consent from all subjects. All subjects gave written informed consent in accordance with the Declaration of Helsinki. The protocol was approved by the University of Illinois at Chicago (UIC) Institutional Review Board.

## Author Contributions

EJ performed research, analyzed and interpreted data, and wrote and edited manuscript. NS analyzed and interpreted data and revised and edited manuscript. XH interpreted data and wrote manuscript. YY analyzed data and revised manuscript. YH performed research and revised manuscript. DW and RM designed study and revised manuscript. ZW designed study, interpreted data, wrote, revised, and edited manuscript.

## Conflict of Interest Statement

The authors declare that the research was conducted in the absence of any commercial or financial relationships that could be construed as a potential conflict of interest.

## References

[B1] Aisiku P. Smith W. R. McClish D. K. Levenson J. L. Penberthy L. T. Roseff S. D. (2009). Comparisons of high versus low emergency department utilizers in sickle cell disease. *Ann. Emerg. Med.* 53 587–593. 10.1016/j.annemergmed.2008.07.050 18926599

[B2] Ballas S. K. (2005). Pain management of sickle cell disease. *Hematol. Oncol. Clin. North Am.* 19 785–802. 10.1016/j.hoc.2005.07.008 16214644

[B3] Ballas S. K. Gupta K. Adams-Graves P. (2012). Sickle cell pain: a critical reappraisal. *Blood* 120 3647–3656. 10.1182/blood-2012-04-383430 22923496

[B4] Baraka M. Darwish I. E. Ghoneim M. T. Korayem H. K. (2015). beta2-adrenoceptor agonists as potential therapeutic drugs in diabetic peripheral neuropathy. *Eur. J. Pharmacol* 746 89–95. 10.1016/j.ejphar.2014.11.004 25445052

[B5] Barrett J. C. Fry B. Maller J. Daly M. J. (2005). Haploview: analysis and visualization of LD and haplotype maps. *Bioinformatics* 21 263–265. 10.1093/bioinformatics/bth457 15297300

[B6] Belfer I. Buzas B. Evans C. Hipp H. Phillips G. Taubman J. (2005). Haplotype structure of the beta adrenergic receptor genes in US Caucasians and African Americans. *Eur. J. Hum. Genet.* 13 341–351. 10.1038/sj.ejhg.5201313 15523499

[B7] Bohren Y. Tessier L. H. Megat S. Petitjean H. Hugel S. Daniel D. (2013). Antidepressants suppress neuropathic pain by a peripheral beta2-adrenoceptor mediated anti-TNFalpha mechanism. *Neurobiol. Dis.* 60 39–50. 10.1016/j.nbd.2013.08.012 23978467

[B8] Brousseau D. C. Owens P. L. Mosso A. L. Panepinto J. A. Steiner C. A. (2010). Acute care utilization and rehospitalizations for sickle cell disease. *JAMA* 303 1288–1294. 10.1001/jama.2010.378 20371788

[B9] Cameron A. C. Trivedi P. K. (1998). *Regression Analysis of Count Data.* Cambridge: Cambridge University Press 10.1017/CBO9780511814365

[B10] Campbell C. M. Carroll C. P. Kiley K. Han D. Haywood C. Jr. Lanzkron S. (2015). Quantitative sensory testing and pain-evoked cytokine reactivity: comparison of patients with sickle cell disease to healthy matched controls. *Pain* 157 949–956. 10.1097/j.pain.0000000000000473 26713424 PMC4932897

[B11] Carroll P. Haywood C. Lanzkron S. (2011). Prediction of onset and course of high hospital utilization in sickle cell disease. *J. Hosp. Med.* 6 248–255. 10.1002/jhm.850 21661098

[B12] Choucair-Jaafar N. Beetz N. Gilsbach R. Yalcin I. Waltisperger E. Freund-Mercier M. J. (2011). Cardiovascular effects of chronic treatment with a beta2-adrenoceptor agonist relieving neuropathic pain in mice. *Neuropharmacology* 61 51–60. 10.1016/j.neuropharm.2011.02.015 21352833

[B13] Choucair-Jaafar N. Salvat E. Freund-Mercier M. J. Barrot M. (2014). The antiallodynic action of nortriptyline and terbutaline is mediated by beta(2) adrenoceptors and delta opioid receptors in the ob/ob model of diabetic polyneuropathy. *Brain Res.* 1546 18–26. 10.1016/j.brainres.2013.12.016 24361988

[B14] Choucair-Jaafar N. Yalcin I. Rodeau J. L. Waltisperger E. Freund-Mercier M. J. Barrot M. (2009). Beta2-adrenoceptor agonists alleviate neuropathic allodynia in mice after chronic treatment. *Br. J. Pharmacol.* 158 1683–1694. 10.1111/j.1476-5381.2009.00510.x 19912227 PMC2801209

[B15] Clarke G. M. Anderson C. A. Pettersson F. H. Cardon L. R. Morris A. P. Zondervan K. T. (2011). Basic statistical analysis in genetic case-control studies. *Nat. Protoc.* 6 121–133. 10.1038/nprot.2010.182 21293453 PMC3154648

[B16] Diatchenko L. Anderson A. D. Slade G. D. Fillingim R. B. Shabalina S. A. Higgins T. J. (2006). Three major haplotypes of the beta2 adrenergic receptor define psychological profile, blood pressure, and the risk for development of a common musculoskeletal pain disorder. *Am. J. Med. Genet. B Neuropsychiatr. Genet.* 141B, 449–462. 10.1002/ajmg.b.30324 16741943 PMC2570772

[B17] Epstein K. Yuen E. Riggio J. M. Ballas S. K. Moleski S. M. (2006). Utilization of the office, hospital and emergency department for adult sickle cell patients: a five-year study. *J. Natl. Med. Assoc.* 98 1109–1113. 16895280 PMC2569470

[B18] Eyler C. E. Jackson T. Elliott L. E. De Castro L. M. Jonassaint J. Ashley-Koch A. (2008). beta(2)-Adrenergic receptor and adenylate cyclase gene polymorphisms affect sickle red cell adhesion. *Br. J. Haematol.* 141 105–108. 10.1111/j.1365-2141.2008.07008.x 18324973

[B19] Ezenwa M. O. Molokie R. E. Wang Z. J. Yao Y. Suarez M. L. Angulo V. (2014). Outpatient pain predicts subsequent one-year acute health care utilization among adults with sickle cell disease. *J. Pain Symptom Manage.* 48 65–74. 10.1016/j.jpainsymman.2013.08.020 24636960 PMC4082743

[B20] Faul F. Erdfelder E. Lang A. G. Buchner A. (2007). G^∗^Power 3: a flexible statistical power analysis program for the social, behavioral, and biomedical sciences. *Behav. Res. Methods* 39 175–191. 10.3758/BF03193146 17695343

[B21] Green S. A. Turki J. Bejarano P. Hall I. P. Liggett S. B. (1995). Influence of beta 2-adrenergic receptor genotypes on signal transduction in human airway smooth muscle cells. *Am. J. Respir. Cell Mol. Biol.* 13 25–33. 10.1165/ajrcmb.13.1.7598936 7598936

[B22] Green S. A. Turki J. Innis M. Liggett S. B. (1994). Amino-terminal polymorphisms of the human beta 2-adrenergic receptor impart distinct agonist-promoted regulatory properties. *Biochemistry* 33 9414–9419. 10.1021/bi00198a006 7915137

[B23] Hein L. (2006). Adrenoceptors and signal transduction in neurons. *Cell Tissue Res.* 326 541–551. 10.1007/s00441-006-0285-2 16896948

[B24] Hocking L. J. Smith B. H. Jones G. T. Reid D. M. Strachan D. P. Macfarlane G. J. (2010). Genetic variation in the beta2-adrenergic receptor but not catecholamine-O-methyltransferase predisposes to chronic pain: results from the 1958 british birth cohort study. *Pain* 149 143–151. 10.1016/j.pain.2010.01.023 20167428

[B25] Jhun E. He Y. Yao Y. Molokie R. E. Wilkie D. J. Wang Z. J. (2014). Dopamine D3 receptor Ser9Gly and catechol-o-methyltransferase Val158Met polymorphisms and acute pain in sickle cell disease. *Anesth. Analg.* 119 1201–1207. 10.1213/ANE.0000000000000382 25102390 PMC4205211

[B26] Jhun E. He Y. Yao Y. Wilkie D. Molokie R. Wang J. (2016). (283) Beta2-adrenergic receptor gene polymorphisms and haplotypes associate with chronic pain in sickle cell disease. *J. Pain* 17 S46–S47. 10.1016/j.jpain.2016.01.189 PMC6390066 30837870

[B27] Lanzkron S. Haywood C. Segal J. B. Dover G. J. (2006). Hospitalization rates and costs of care of patients with sickle-cell anemia in the state of Maryland in the era of hydroxyurea. *Am. J. Hematol.* 81 927–932. 10.1002/ajh.20703 16924648

[B28] Lanzkron S. Little J. Field J. Shows J. R. Wang H. Seufert R. (2018). Increased acute care utilization in a prospective cohort of adults with sickle cell disease. *Blood Adv.* 2 2412–2417. 10.1182/bloodadvances.2018018382 30254105 PMC6156885

[B29] Lettre G. Lange C. Hirschhorn J. N. (2007). Genetic model testing and statistical power in population-based association studies of quantitative traits. *Genet. Epidemiol.* 31 358–362. 10.1002/gepi.20217 17352422

[B30] Liang D. Y. Shi X. Li X. Li J. Clark J. D. (2007). The beta2 adrenergic receptor regulates morphine tolerance and physical dependence. *Behav. Brain Res.* 181 118–126. 10.1016/j.bbr.2007.03.037 17498818 PMC1989675

[B31] Litonjua A. Gong L. Duan Q. L. Shin J. Moore M. J. Weiss S. T. (2010). Very important pharmacogene summary ADRB2. *Pharmacogenet. Genom.* 20 64–69. 10.1097/FPC.0b013e328333dae6 19927042 PMC3098753

[B32] Melzack R. (1975). The McGill pain questionnaire: major properties and scoring methods. *Pain* 1 277–299. 10.1016/0304-3959(75)90044-5 1235985

[B33] Nicholson R. Dixon A. K. Spanswick D. Lee K. (2005). Noradrenergic receptor mRNA expression in adult rat superficial dorsal horn and dorsal root ganglion neurons. *Neurosci. Lett.* 380 316–321. 10.1016/j.neulet.2005.01.079 15862909

[B34] Peng Y. I. Liu H. J. Fu T. C. (1993). Involvement of alpha- and beta-adrenoceptors in antinociception at the lumber spinal level in mice. *Chin. J. Physiol.* 36 177–180. 8194393

[B35] Platt O. S. Thorington B. D. Brambilla D. J. Milner P. F. Rosse W. F. Vichinsky E. (1991). Pain in sickle cell disease. Rates and risk factors. *N. Engl. J. Med.* 325 11–16. 10.1056/NEJM199107043250103 1710777

[B36] Purcell S. (2010). *PLINK version 1.07.*

[B37] Purcell S. Neale B. Todd-Brown K. Thomas L. Ferreira M. A. Bender D. (2007). PLINK: a tool set for whole-genome association and population-based linkage analyses. *Am. J. Hum. Genet.* 81 559–575. 10.1086/519795 17701901 PMC1950838

[B38] Rees D. C. Williams T. N. Gladwin M. T. (2010). Sickle-cell disease. *Lancet* 376 2018–2031. 10.1016/S0140-6736(10)61029-X 21131035

[B39] Reiner S. Ambrosio M. Hoffmann C. Lohse M. J. (2010). Differential signaling of the endogenous agonists at the beta2-adrenergic receptor. *J. Biol. Chem.* 285 36188–36198. 10.1074/jbc.M110.175604 20837485 PMC2975241

[B40] Saadi V. Gupta H. Angural A. Dhanya S. K. Mony S. Oberoi D. (2013). Single nucleotide polymorphisms of ADRB2 gene and their association with susceptibility for Plasmodium falciparum malaria and asthma in an Indian population. *Infect. Genet. Evol.* 20 140–147. 10.1016/j.meegid.2013.08.026 24012958

[B41] Sadhu N. Jhun E. H. Yao Y. He Y. Molokie R. E. Wilkie D. J. (2018). Genetic variants of GCH1 associate with chronic and acute crisis pain in African Americans with sickle cell disease. *Exp. Hematol.* 66 42–49. 10.1016/j.exphem.2018.07.004 30031848 PMC6175655

[B42] Samoshkin A. Convertino M. Viet C. T. Wieskopf J. S. Kambur O. Marcovitz J. (2015). Structural and functional interactions between six-transmembrane mu-opioid receptors and beta2-adrenoreceptors modulate opioid signaling. *Sci. Rep.* 5:18198. 10.1038/srep18198 26657998 PMC4676002

[B43] Schurks M. Kurth T. Ridker P. M. Buring J. E. Zee R. Y. (2009). Association between polymorphisms in the beta2-adrenoceptor gene and migraine in women. *Headache* 49 235–244. 10.1111/j.1526-4610.2008.01207.x 18647184 PMC2644736

[B44] Shankar S. M. Arbogast P. G. Mitchel E. Cooper W. O. Wang W. C. Griffin M. R. (2005). Medical care utilization and mortality in sickle cell disease: a population-based study. *Am. J. Hematol.* 80 262–270. 10.1002/ajh.20485 16315251

[B45] Skouen J. S. Smith A. J. Warrington N. M. O’ Sullivan P. B. McKenzie L. Pennell C. E. (2012). Genetic variation in the beta-2 adrenergic receptor is associated with chronic musculoskeletal complaints in adolescents. *Eur. J. Pain* 16 1232–1242. 10.1002/j.1532-2149.2012.00131.x 22416031

[B46] Small K. M. McGraw D. W. Liggett S. B. (2003). Pharmacology and physiology of human adrenergic receptor polymorphisms. *Annu. Rev. Pharmacol. Toxicol.* 43 381–411. 10.1146/annurev.pharmtox.43.100901.135823 12540746

[B47] Smith W. R. Penberthy L. T. Bovbjerg V. E. McClish D. K. Roberts J. D. Dahman B. (2008). Daily assessment of pain in adults with sickle cell disease. *Ann. Intern. Med.* 148 94–101. 10.7326/0003-4819-148-2-200801150-00004 18195334

[B48] Todd J. (2010). Neuronal circuitry for pain processing in the dorsal horn. *Nat. Rev. Neurosci.* 11 823–836. 10.1038/nrn2947 21068766 PMC3277941

[B49] Torjussen T. M. Munthe-Kaas M. C. Mowinckel P. Carlsen K. H. Undlien D. E. Lodrup Carlsen K. C. (2013). Childhood lung function and the association with beta2-adrenergic receptor haplotypes. *Acta Paediatr.* 102 727–731. 10.1111/apa.12221 23463918

[B50] Vargas-Alarcon G. Fragoso J. M. Cruz-Robles D. Vargas A. Martinez A. Lao-Villadoniga J. I. (2009). Association of adrenergic receptor gene polymorphisms with different fibromyalgia syndrome domains. *Arthritis Rheum* 60 2169–2173. 10.1002/art.24655 19565482

[B51] Wang H. Heijnen C. J. Eijkelkamp N. Garza Carbajal A. Schedlowski M. Kelley K. W. (2011). GRK2 in sensory neurons regulates epinephrine-induced signalling and duration of mechanical hyperalgesia. *Pain* 152 1649–1658. 10.1016/j.pain.2011.03.010 21514055

[B52] Wang S. Zhu H. Y. Jin Y. Zhou Y. Hu S. Liu T. (2015). Adrenergic signaling mediates mechanical hyperalgesia through activation of P2X3 receptors in primary sensory neurons of rats with chronic pancreatitis. *Am. J. Physiol. Gastrointest. Liver Physiol.* 308 G710–G719. 10.1152/ajpgi.00395.2014 25634810

[B53] Wilkie D. J. Judge M. K. Berry D. L. Dell J. Zong S. Gilespie R. (2003). Usability of a computerized PAINReportIt in the general public with pain and people with cancer pain. *J. Pain Symptom Manage.* 25 213–224. 10.1016/S0885-3924(02)00638-3 12614956

[B54] Wilkie D. J. Molokie R. Boyd-Seal D. Suarez M. L. Kim Y. O. Zong S. (2010). Patient-reported outcomes: descriptors of nociceptive and neuropathic pain and barriers to effective pain management in adult outpatients with sickle cell disease. *J. Natl. Med. Assoc.* 102 18–27. 10.1016/S0027-9684(15)30471-5 20158132 PMC3641147

[B55] Wilkie D. J. Molokie R. E. Suarez M. L. Ezenwa M. O. Wang Z. J. (2015). Composite pain index: reliability, validity, and sensitivity of a patient-reported outcome for research. *Pain Med.* 16 1341–1348. 10.1111/pme.12703 25712169 PMC4504760

[B56] Yalcin I. Tessier L. H. Petit-Demouliere N. Waltisperger E. Hein L. Freund-Mercier M. J. (2010). Chronic treatment with agonists of beta(2)-adrenergic receptors in neuropathic pain. *Exp. Neurol.* 221 115–121. 10.1016/j.expneurol.2009.10.008 19840789

[B57] Zhang C. Rui Y. Y. Zhou Y. Y. Ju Z. Zhang H. H. Hu C. Y. (2014). Adrenergic beta2-receptors mediates visceral hypersensitivity induced by heterotypic intermittent stress in rats. *PLoS One* 9:e94726. 10.1371/journal.pone.0094726 24733123 PMC3986230

[B58] Zhang H. T. Huang Y. O’Donnell J. M. (2003). Antagonism of the antidepressant-like effects of clenbuterol by central administration of beta-adrenergic antagonists in rats. *Psychopharmacology* 170 102–107. 10.1007/s00213-003-1512-0 12898120

[B59] Zhou J. Han J. Nutescu E. A. Gordeuk V. R. Saraf S. L. Calip G. S. (2018). Hydroxycarbamide adherence and cumulative dose associated with hospital readmission in sickle cell disease: a 6-year population-based cohort study. *Br. J. Haematol.* 182 259–270. 10.1111/bjh.15396 29767446 PMC6037608

